# 46. Templated Microbiology Comments with Candiduria to Enhance Antimicrobial Stewardship

**DOI:** 10.1093/ofid/ofab466.248

**Published:** 2021-12-04

**Authors:** Weston Schartz, Nick Bennett, Laura Aragon, Kevin Kennedy, Sarah E Boyd, Matthew Humphrey, Cindy Essmyer

**Affiliations:** Saint Luke’s Health System, Blue springs, Missouri

## Abstract

**Background:**

Behavioral interventions have been shown to improve antimicrobial selection. Such practices are low cost and effective means of stewardship promotion. One area of overtreatment that contributes to unnecessary antifungal use is in hospitalized patients with candiduria. We implemented a templated microbiology comment to guide prescribing of antifungals for hospitalized patients with candiduria.

**Methods:**

This was a quasi-experimental, multi-center, single health system study. When *Candida* is isolated, the following comment appears in the microbiology result section along with the urine culture result: “In the absence of symptoms, *Candida* is generally considered normal flora. No therapy indicated unless high risk (pregnant, neonate or neutropenic) or undergoing urologic procedure. If Foley catheter present, remove or replace when able.” We compared a pre-implementation cohort (June 2018-Janurary 2019) to a post-implementation cohort (June 2019-Janurary 2020). Patients were included in the study if they were inpatients, 18 years and older, with candiduria. The primary outcome was the rate of antifungal administration within 72 hours after culture results became available. Secondary outcomes include duration of therapy and rate of antifungal given within 73-240 hours after culture result.

**Results:**

The study included a total of 297 patients between the two groups (156 pre-implementation, 141 post-implementation). The primary outcome was found to be significantly lower in the post-implementation group (48.1% vs 34.0%, p=0.014). A multivariate adjustment for baseline characteristics that were significantly different between groups revealed that post-implementation group maintained its effect (OR 0.49 (0.29, 0.82), p=0.0067). For secondary outcomes, no difference was found in patients requiring antifungal administration within 73-240 hours after microbiology results were available (1.3% vs 3.5%, p=0.199). There was no difference in mean antifungal duration (4 vs 3 days, p=0.449).

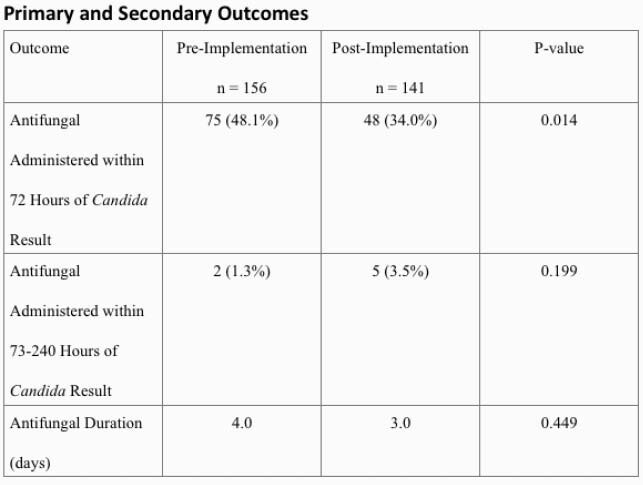

**Conclusion:**

Adding a templated comment to urine cultures was associated with a significant reduction in the number of antifungals prescribed in patients with candiduria. This strategy is an effective low-cost, passive education technique to improve antimicrobial stewardship.

**Disclosures:**

**All Authors**: No reported disclosures

